# Estimating lifetime healthcare costs with morbidity data

**DOI:** 10.1186/1472-6963-13-440

**Published:** 2013-10-25

**Authors:** Marc Carreras, Pere Ibern, Jordi Coderch, Inma Sánchez, Jose M Inoriza

**Affiliations:** 1 GRESSiRES, Research group on health services and health outcomes, Serveis de Salut Integrats Baix Empordà, Hospital 27, Palamós, 17230, Spain; 2 Departament d’Empresa, Universitat de Girona, Campus de Montilivi, Girona, 17071, Spain; 3 Barcelona Graduate School of Economics, Ramon Trias Fargas 25-27, Barcelona, 08005, Spain

**Keywords:** Lifetime healthcare costs, Chronic disease, Clinical Risk Groups, Markov Chain

## Abstract

**Background:**

In many developed countries, the economic crisis started in 2008 producing a serious contraction of the financial resources spent on healthcare. Identifying which individuals will require more resources and the moment in their lives these resources have to be allocated becomes essential. It is well known that a small number of individuals with complex healthcare needs consume a high percentage of health expenditures. Conversely, little is known on how morbidity evolves throughout life. The aim of this study is to introduce a longitudinal perspective to chronic disease management.

**Methods:**

Data used relate to the population of the county of Baix Empordà in Catalonia for the period 2004–2007 (average population was N = 88,858). The database included individual information on morbidity, resource consumption, costs and activity records. The population was classified using the Clinical Risk Groups (CRG) model. Future morbidity evolution was simulated under different assumptions using a stationary Markov chain. We obtained morbidity patterns for the lifetime and the distribution function of the random variable lifetime costs. Individual information on acute episodes, chronic conditions and multimorbidity patterns were included in the model.

**Results:**

The probability of having a specific health status in the future (healthy, acute process or different combinations of chronic illness) and the distribution function of healthcare costs for the individual lifetime were obtained for the sample population. The mean lifetime cost for women was €111,936, a third higher than for men, at €81,566 (all amounts calculated in 2007 Euros). Healthy life expectancy at birth for females was 46.99, lower than for males (50.22). Females also spent 28.41 years of life suffering from some type of chronic disease, a longer period than men (21.9).

**Conclusions:**

Future morbidity and whole population costs can be reasonably predicted, combining stochastic microsimulation with a morbidity classification system. Potential ways of efficiency arose by introducing a time perspective to chronic disease management.

## Background

In many developed countries, the economic crisis started in 2008 producing a serious contraction of the financial resources spent on healthcare. Moreover, demographic development in recent decades has resulted in older populations which require more resources and may generate higher expenditures [1,2]. This unfavourable context increases the concern for refining cost estimates and resource allocation [3]. Under such a scenario, identifying which individuals will require more resources and the moment in their lives these resources have to be allocated becomes essential.

It is well known that a small number of individuals with complex healthcare needs consume a high percentage of health expenditures [4,5]. Furthermore, for demographic reasons, from the decade of 90s, healthcare systems were faced with an increasing impact of chronic disease and multimorbidity. As a response, integrated practice and chronic disease management arose as a first effort for maximizing efficiency. The stratification of the population according to their risks is a fundamental part of such framework, being individuals and their related medical conditions the key elements for the allocation of resources [6,7].

Conversely, a little is known on how morbidity evolves throughout life. Although recent studies suggest the importance of adopting a life cycle perspective [8], research in health economics has only started to consider how life conditions observed at a specific point in time influence health in the future [9-11]. A sound knowledge on how individual and policy decisions influences transitions between health states during life, especially in the early years of life, could bring new opportunities for maximizing effectiveness and avoiding costs.

According to the literature, healthcare costs and utilization over the life time can be obtained from cross sectional studies. For example, risk assessment models obtain the whole population characteristics by mixing different cohorts at a point in time [12]. However, the life history of a particular individual or the variability among individuals included in the same age-gender cell is not disclosed in cross-sectional studies.

The alternative is the longitudinal analysis. The Canadian population health model POHEM provides risk assessment throughout the life span for people with specific chronic diseases. e.g. different types of cancer, osteoarthritis, acute myocardial infarction, diabetes and for disease risk factors such as obesity and physical inactivity [13]. Longitudinal studies occasionally include costs, which can be related to the economic evaluation of specific technologies [14]. Other longitudinal studies simulate future expenditures for a particular cohort using specific mortality and population projections [1,2]. The work of Alemayehu and Warner is particularly original, which models cross-sectional expenditures under the mortality experience obtained from a period life table [15]. Moreover, Forget et al. apply Markov modelling to potential future expenditures under a cost categories transition scheme [3]. Unfortunately, any of the above models consider a comprehensive perspective for the individual morbidity or health state. Only the population model POPMOD develops a multi-state model for two dependent interacting disease conditions and mortality [16].

Recent studies go further by proposing a joint analysis for the main chronic diseases [17,18]. Our work aims to follow this approach, but from a different perspective. Thinking in how actual health conditions and decisions can affect health in the future, we improved the article of Forget et al. [3], including health states in the simulation. Individual information on acute episodes, chronic conditions and multimorbidity has been summarized for a specific population using a classification system for risk-adjustment population-based payment: *Clinical Risk Groups* (CRG) [19]. We obtained morbidity patterns for the lifetime and the distribution function of the random variable lifetime costs, considering age and gender, but including at the same time chronic conditions and multimorbidity.

A number of studies describe health states and consumption patterns by gender [20,21], at different moments of life [22-24], or for a specific chronic disease [13]. However, with few exceptions [25], it is difficult to find studies that describe a complete pattern for the whole life. In this article we try to fill this gap. *The aim of this study is to introduce a longitudinal perspective to chronic disease management*. From a public health point of view, a sound knowledge on health states transitions would benefit healthcare planners, which could promote prevention and actions over specific groups of population at risk. Moreover, healthcare providers involved in integrated practice and chronic disease management, could avoid future costs by focusing on those individuals who are susceptible to suffer a deterioration on their health state.

## Methods

### Data

Data used relate to the population of the county of Baix Empordà in Catalonia for the period 2004–2007. The average population was N = 88,858. Natural population movements and migrations (new arrivals and departures) were continuously recorded. For our purposes, yearly counts of individuals were made at the end of each year.

The information was provided by *Serveis de Salut Integrats del Baix Empordà* (SSIBE), an integrated healthcare management organisation responsible for providing public healthcare services, including primary care, specialised care, acute hospitalisations and long-term residential care. For administration purposes SSIBE runs an integrated patient database with clinical records and individual information on use of resources and activity for the whole population [26].

Using the CRG model, individuals were classified into single, mutually exclusive, and exhaustive categories according to their clinical findings and demographic characteristics. The original CRG core health status classification aggregates individual morbidity histories into nine categories: (1) Healthy^a^, (2) History of significant acute disease, (3) Single minor chronic disease, (4) Minor chronic disease in multiple organ systems, (5) Single dominant or moderate chronic disease, (6) Significant chronic disease in multiple organ system, (7) Dominant chronic disease in three or more organ systems, (8) Dominant and metastatic malignancies and (9) Catastrophic conditions. However, in order to avoid CRG cells grouping a small number of individuals, we aggregated individual morbidity histories into six core health status, following a classification based on the article of Neff et al. [27]: E1 Healthy, E2 Significant acute disease, E34 Minor chronic disease, E56 Significant chronic disease in one or two organ systems, E79 Significant chronic disease in three or more organ systems - Catastrophic conditions, E8 Dominant and metastatic malignancies [28].

Individual morbidity categories (CRG) were generated every year using all the individual information on diagnostics and procedures related to the population. For example, the system processed 857,385 ICD codes (815,227 diagnostics and 42,158 procedures collected between 1/1/2007 and 31/12/2007) that were related to the 90,595 individuals included in the study in 2007.

The demographic characteristics and the risk profile of the population in 2007 are shown in Tables 1 and 2 respectively. Males made up a slightly larger group, and about 65% of the SSIBE population belonged to the status *Healthy*.

**Table 1:** Demographic characteristics of the population at 1/1/2007

***N***	**90,595**
Female	49.50%
Male	50.50%
Age (mean)	39.48
0-1	1.08%
1-14	14.35%
15-24	11.17%
25-34	17.78%
35-44	16.86%
45-54	12.90%
55-64	9.76%
64-74	7.91%
75-84	6.12%
85 or older	2.07%

**Table 2:** Aggregated clinical risk group categories of patients at 1/1/2007

***Core Health Status Groups***	***%***
E1. Healthy	65.45%
E2. Significant acute disease	9.05%
E34. Minor chronic disease	7.43%
E56. Significant chronic disease in one or two organ systems	17.26%
E79. Significant chronic disease in three or more organ systems - Catastrophic conditions	0.58%
E8. Dominant and metastatic malignancies	0.23%

### Ethics

This study is part of a research on population morbidity and healthcare expenditures and was carried out using individual anonymous data provided by SSIBE. The Research Committee of SSIBE authorized the study and the data transfer protocol.

### Morbidity patterns for the lifetime

Individual morbidity patterns for the lifetime were obtained by assuming they follow a stationary Markov chain. A Markov chain is defined as a discrete stochastic process which meets the Markov property. Suppose that time takes the discrete values *t = 1,2, …, n* (natural years in our case), and for any given time, the health state X_t_ can take the value *x*_*t*_*,* from the discrete space of states *x*_*t,1*_*,x*_*t,2*_*,…,x*_*t,m*_. Since there are six core CRG health status groups, and death is an additional, absorbing, state, we have *m =* 7). The Markov property then is defined as:

$$\begin{array}{l} P\left(X_{t}=x_{t}\left|X_{t-1}=x_{t-1}\right),\ldots ,X_{2}=x_{2},X_{1}=x_{1}\right)\\ \;=P\left(X_{t}=x_{t}\left|X_{t-1}=x_{t-1}\right)\right), \end{array}$$

the fundamental assumption is that the health state of an individual for a specific year depended only on the health state observed the year before. Such characteristics correspond to the Markov Chain framework [29-32].

The transition probabilities in the Markov model were easily inferred because the complete health state sequence (from 2004 to 2007) was known for the entire population. Therefore, transition probabilities were obtained by observing individual status changes for each pair of years in the study (2004–2005, 2005–2006, 2006–2007) as simple counts or frequencies, using the maximum likelihood estimator:

$${\hat{p}}_{\mathrm{ij}}=\sum_{t=1}^{T}n_{\mathrm{ij}}\left(t\right)/\sum_{z=1}^{m}\;\sum_{t-1}^{T}n_{\mathrm{iz}}\left(t\right),$$

were *t = 1, 2, …, T* are the times of observation, *i, j = 1, 2, …, m* are the states of the process and *n*_*ij*_ are the number of individuals in state *j* at time *t*, having state *i* at time *t-1*.

The probability estimates were obtained from the data according to the formula. Twenty transition matrices were obtained, considering gender and ten age groups: < 1 year of age, 1–14 years of age, 15–24, 25–34, 35–44, 45–54, 55–64, 65–74, 75–84, ≥ 85.

In the above we have assumed stationarity in the Markov chain. Consequently, transition probabilities are supposed to be stationary or homogeneous, i.e. transition matrices are considered stable, and the probability of moving from one specific health state to another remains constant over time. The adequacy of the stationary property was tested by comparing distances between stationary and temporal probabilities to the *X*_*i*_ squared distribution with *m(m-1)(T-1)* degrees of freedom:

$$\frac{\sum_{t}\sum_{t,j}n_{i}\left(t‒1\right){\left[{\hat{p}}_{\mathrm{ij}}\left(t\right)‒{\hat{p}}_{\mathrm{ij}}\right]}^{2}}{{\hat{p}}_{\mathrm{ij}}}\Rightarrow X_{m\left(m-1\right)\left(T-1\right)}^{2}$$

Test results showed how all the probabilities included in transition matrices were stationary during the period 2004–2007, with the exception of men aged 75–84.

The morbidity evolution was obtained from the Markov chain structure by combining two simulation strategies:

1. Markov cohort simulation: A cohort of N = 100,000 individuals, evolved from birth to death. The initial cohort composition followed the morbidity and gender structure of the 2007 population (0–1 years of age group). Then, the simulation algorithm generated a sequence of cycles until the complete extinction of the cohort. A new cycle in the simulation reproduced a year of life for the cohort: individuals which were grouped according to their gender, age and health state characteristics, changed their health conditions according to the appropriate transition probabilities.

2. Monte Carlo simulation: The second simulation generated random lifetime health histories. The process started assigning a random health status for a standard individual. Starting at birth, the simulation algorithm generated a sequence of cycles until his death. A new cycle in the simulation reproduced a new year of life for such individual. Changes in health status during life were obtained generating pseudo-random numbers and comparing them to the transition probabilities. The process was repeated for N_1_ = 10,000 females and N_2_ = 10,000 males. Finally, the result was a collection of pathways.

These strategies were complementary. The result of the first simulation was exact, according to the initial cohort characteristics and the transition matrix of probabilities. Conversely, depending on the number of trials, the result of the second simulation may contain certain bias as a consequence of the use of pseudo-random numbers. The major advantage of the second simulation is that it provides measures of variability.

### Adding costs to the model

Once the projection of the future population morbidity was obtained, the next step in the process involved the evaluation of costs. Depending on the simulation strategy, a new cycle generated a new number of individuals with specific age, gender and health status (a new year in the cohort history) or a new specific individual condition (a new year in the individual health history). Throughout the process, we evaluated costs for these situations using average costs adjusted by age, gender and CRG status. Mean cost estimates corresponded to the SSIBE database from year 2007.

Table 3 summarizes mean costs by gender and health status for the whole population in 2007. Mean costs included primary care, specialised care, acute hospitalisations, long-term residential care and pharmaceutical consumption outside the institution.

**Table 3:** Cost (euros) by health status

***Core Health Status Groups***	***Mean cost***	
	***Females***	***Males***
E1. Healthy	324.16	227.42
E2. Significant acute disease	1,692.67	1,195.57
E34. Minor chronic disease	1,069.41	936.65
E56. Significant chronic disease in one or two organ systems	2,739.67	2,334.04
E79. Significant chronic disease in three or more organ systems - Catastrophic conditions	11,095.93	10,113.71
E8. Dominant and metastatic malignancies	5,617.47	5,519.98

Year 2007.

The SSIBE costing methodology is a retrospective full-cost system [33], which combines the bottom-up and the top-down approaches described in [34]. Therefore, direct costs (blood transfusions, prostheses, intermediate products, and pharmaceutical consumption) were directly attributed to patients from their clinical records using the bottom-up approach. At the same time, using activity records and the related unit costs, departmental costs were apportioned to patients according to the individual consumption of health services resources (top-down microcosting approach).

It is important to remember that SSIBE is responsible for the public provision of first-level healthcare: I.e. complex care - for example organ transplant, neonatology or neurosurgery - is systematically referred to complex hospitals outside the county. External referrals represented 2.2% of the total activity and 17% of the total costs for SSIBE in 2007 [35]. The main cases of external referrals included high-complexity hospitalisations, psychiatric hospitalisation and ambulatory mental health.

## Results and discussion

### Cohort simulation

**Figure 1:**
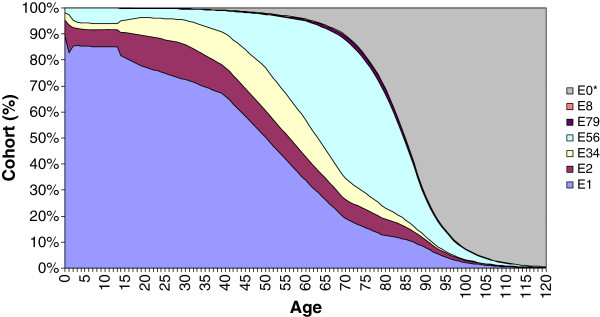
**Cohort simulation - Females.** E1: Healthy. E2: Significant acute disease. E34: Minor chronic disease. E56: Significant chronic disease in one or two organ systems. E79: Significant chronic disease in three or more organ systems - Catastrophic conditions. E8: Dominant and metastatic malignancies. E0*: Death (Added as the absorbing state).

**Figure 2:**
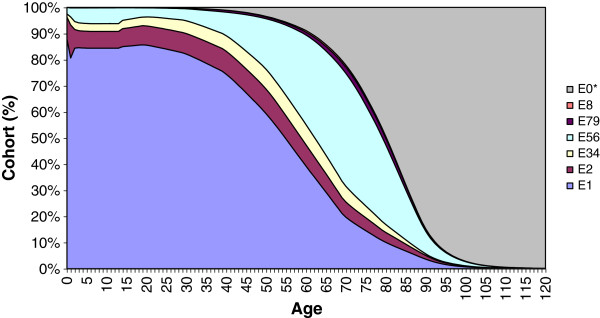
**Cohort simulation - Males.** E1: Healthy. E2: Significant acute disease. E34: Minor chronic disease. E56: Significant chronic disease in one or two organ systems. E79: Significant chronic disease in three or more organ systems - Catastrophic conditions. E8: Dominant and metastatic malignancies. E0*: Death (Added as the absorbing state).

The result of the first simulation was a morbidity projection for a cohort of N = 100,000 individuals. Since the time horizon was over the whole life cycle, people developed from birth to death. We obtained the probability of having a specific health state in the future. Figures 1 and 2 show the lifetime burden of morbidity by gender at any age.

The first years of life were particularly complex. Figures 1 and 2 above show abrupt probability jumps due to age group construction (< 1 year of age, 1–14 years of age). Approximately 96% of the initial cohort was classified as healthy individuals. Nevertheless, Figures 1 and 2 show a serious peak of acute processes related to different health problems in the first year of life.

Gender differences were remarkable through life. In general, with the exception of the early age groups (< 1 year of age, 1–14 years of age), the burden of morbidity was higher for females. Women clearly had a higher probability of suffering an acute process as a consequence of childbearing age (14–40 years of age)^b^. Moreover, the healthy status area was proportionally reduced (see Figures 1 and 2). The situation after this period did not improve for women, the healthy status area continued to be narrower than for men, with an increase in the main chronic categories. Conversely, the yearly probabilities of having a healthy life were higher for males above 14 years of age. At the same time they experienced a proportional reduction in the probabilities of falling into a chronic category.

Table 4 shows expected years at birth by gender and health status.

**Table 4:** Expected years of life at birth by health status (individual simulation)

***Core health status groups***		***Females***					***Males***			
		***Mean***	***SD***	***Min***	***Max***		***Mean***	***SD***	***Min***	***Max***
E1. Healthy	**-**	*46.99*	7.26	2	80	**+**	*50.22*	7.25	17	81
E2. Significant acute disease	**+**	*8.39*	3.37	0	24	**-**	*6.30*	2.92	0	20
E34. Minor chronic disease	**+**	*7.74*	3.63	0	26	**-**	*4.37*	2.69	0	20
E56. Significant chronic disease in one or two organ systems	**+**	*19.96*	8.40	0	56	**-**	*16.36*	8.31	0	61
E79. Significant chronic disease in three or more organ systems - Catastrophic conditions		0.52	1.09	0	13		0.87	1.65	0	20
E8. Dominant and metastatic malignancies		0.19	0.64	0	9		0.29	0.68	0	8
Total		83.79	-	-	-		78.41	-	-	-

Life expectancy at birth obtained from the model was 83.79 years for females, approximately 5.38 years longer than for males (78.41), and consistent with official public estimates for 2007 [36]. Healthy life expectancy at birth for females was 46.99, lower than for males (50.22). Females also spent 28.41 years of life suffering from some type of chronic disease, a longer period than males (21.9). Finally, males were more likely to fall into the highest morbidity groups: 'E79 Significant chronic disease in three or more organ systems - dominant and metastatic malignancies’ and 'E8 Catastrophic conditions’. However, a small number of individuals were included in both categories. During the period of study, the percentage of population included in categories E79 or E8 remained below 1%.

**Figure 3:**
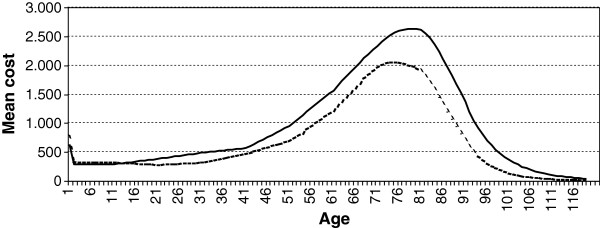
**Yearly mean cost (€).** Females: Continuous line. Males: Dotted line.

A projection of costs was obtained by combining the cohort simulation results with SSIBE costs. Different summations of the Markov Chain generated different results: First, after aggregating costs for a specific year in the projection, we obtained the mean cost by gender (Figure 3). Second, after aggregating the process through life, we obtained the mean lifetime cost for a typical individual. Mean lifetime costs for women was €111,935.96, a third more than for men, at €81,565.67. The relationship between mean costs by gender is consistent with the results from different studies with different populations, in the sense that women use of healthcare resources was a third more than men throughout their lives [3,15].

### Monte Carlo simulation

A collection of pathways were generated from the second simulation. N_1_ = 10,000 for women and N_2_ = 10,000 for men. Each pathway was randomly obtained from observed transitions and represented the lifetime morbidity history for a typical individual. Standard individuals received an initial health status that was generated randomly according to the population morbidity structure from the first age group (< 1 year of age).

The distribution function of the random variable lifetime healthcare costs was drawn from the second simulation. Since we used mean costs by gender, sex and health status, these cumulative frequencies represented primarily the variability of health transitions among individuals. Table 5 shows the main statistics.

**Table 5:** Distribution function of lifetime healthcare cost

	***Females***	***Males***
Number of trials	10,000	10,000
Mean	111,255.10	81,495.67
SD	38,895.93	34,755.81
Variance	1,512,893,414	1,207,966,142
Skewness	0.647	0.491
Kurtosis	2.006	0.844
Minimum	1,071.30	5,757.79
Maximum	446,349.61	334,886.48
*Percentile*		
25	87,820.46	56,629.19
50	108,485.44	81,263.67
75	131,655.66	102,511.78

The mean lifetime healthcare cost obtained from the second simulation was €111,255.10 for women and €81,495.67 for men. Differences in cost averages between both simulation strategies pointed out the bias of the individual simulation. However, such differences remained below 0.7%.

**Figure 4:**
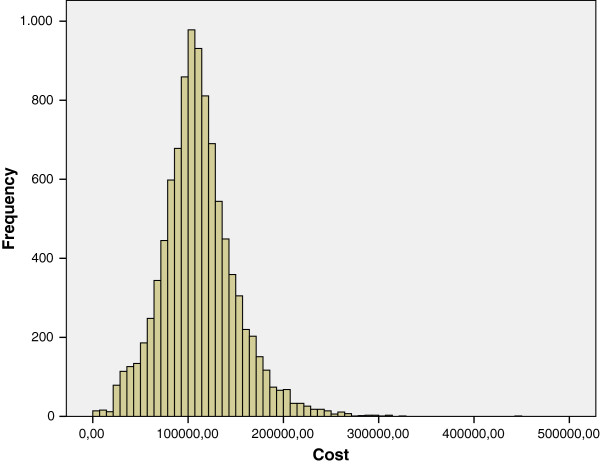
Lifetime healthcare cost distribution (€) - Females.

**Figure 5:**
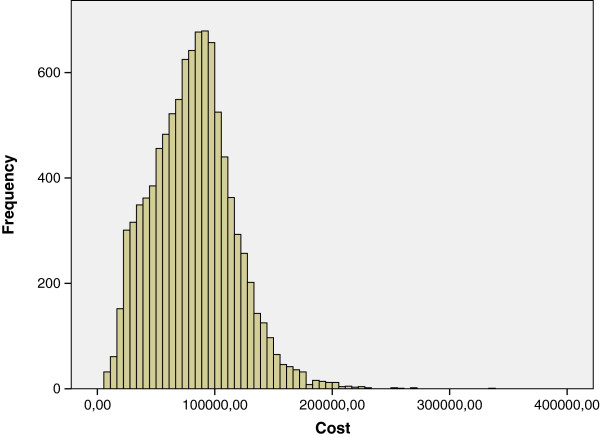
Lifetime healthcare cost distribution (€) - Males.

On the other hand, the distribution of costs of both genders was right-skewed. The higher variance and kurtosis for women can be interpreted as more variability and heavier tails for the distribution of probability, which expressed the heterogeneity of female morbidity (Figures 4 and 5). In other words, the burden of morbidity generated higher costs for women, with more differences among individuals and more individuals with large costs. Such differences in average costs between genders are maintained for percentiles 25th, 50th and 75th.

### Implications

Consistency with other lifetime cost studies and official life expectancy reports imply that the results obtained are reasonable [3,15,36]. But, how can these results be converted in gains in effectiveness and cost reductions? Chronic disease management strategies are based on the stratification of the population according to their risks [4]. Introducing the longitudinal perspective, we observed how individual stocks of health were progressively lost during life, by the impact of acute episodes and by the advance of chronic diseases. Therefore, such information on health transitions could be used to design specific measures for different segments of the population. E.g. healthy individuals or people with complex healthcare needs require distinct measures for preventing health deterioration.

Healthy individuals represent an important share of the population at any age. Considering results from Tables 1, 2 and 3, it is important to see how the amount of healthcare resources spent on healthy individuals largely exceeded the amount spent on individuals with acute hospitalisations^a^. Hughes et al. obtained a similar result using a sample of 1.3 million beneficiaries from Medicare, Medicaid and private insurance [19]. Therefore, healthcare planners should explore specific programs for healthy individuals. For example, promoting healthy lifestyle education, personal responsibility on health or risk prevention in the workplace. Moreover, from a lifetime perspective, reducing transitions from health to worst conditions could generate large health gains for the population and large resource savings for the healthcare system.

According to Figures 1, 2 and 3, from the age group 35–44, the level of mean costs by gender for all ages is strongly related to the amount of individuals with more than one chronic disease. Despite of other effects, for example the amount of individuals with acute hospitalisations. In fact, an important number of individuals suffer a significant chronic disease in one or two organ systems many years during their life (19.96 years females, 16.36 males). Moreover, a small number of patients with a significant chronic disease in three or more organ systems are associated with a significant level of healthcare expenditures. Regarding the high impact of chronic disease during life, chronic disease management strategies offer a good opportunity to gain efficiency by providing cost-effective care to patients with complex care needs [4,5].

From a gender perspective, a higher lifetime healthcare cost for women - a third more than men throughout their lives - is a consequence of a higher life expectancy and a higher acute and chronic disease prevalence. Gender specific measures have a high margin for gaining efficiency.

Finally, specific interventions, including those affecting healthy population, should be implemented following cost-effectiveness standards.

### Model limitations and future research

Although this article is not method-oriented, in the sense that we did not make any essential contribution to the Markov literature, some questions on the fundamental assumptions must be considered.

A first concern is if the Markov property is suitable for describing the individual morbidity development. The fundamental Markov property establishes that the health status of an individual within a specific year depends on the health status the year before. Therefore, did the last twelve month sequence of ICD codes completely describe individual health histories? For a number of reasons, significant ICD codes could be missing in the latest clinical records. Consequently, the CRG classification can slightly underestimate health status, especially for health status E34 and E56^a^. It is important to be careful at this point: CRG categories describe individual behaviour in relation to the use of health services and must be considered an approximation to the health state of individuals. Moreover, due to the unexpected nature of a considerable part of health related events, the maximum predictive capacity for any risk-adjustment system is limited. For risk adjustment purposes, the CRG manual recommends collecting codes for a period of between six months and two years [28]. However, Hughes et al. did not find significant increases in the amount of variance explained by the model using two years of ICD codes instead of one [19].

We made a second important assumption: the Markov chain is stationary. A stationary process is independent from the point in time in which it is observed. In other words, process parameters - transition probabilities in our model - are considered constant over time. According to the results, with the exception of men aged 75–84, transition probabilities are stationary in the period 2004–2007. However, is it reasonable to assume stationary probabilities in the long run? Obviously the answer is no. During the period 1998–2007 life expectancy at birth for the Catalan population increased by 2.12 years [36]. Therefore, transitions to death are not stationary. Moreover, the effect on transitions between health states is unknown. Recent studies suggest that changes in practice and new technologies have produced a demographic change, increasing life expectancy and improving the quality of life. The consequence is a change in patient profiles with an increase in the prevalence of chronic conditions and polypathology. Thorpe and Howard found evidence of the increased prevalence for the top ten chronic conditions (Heart disease, Mental disorders, Trauma, Arthritis, Hypertension, Cancer, Diabetes, Pulmonary conditions, Hyperlipidaemia and Cerebrovascular disease) for US Medicare beneficiaries from 1987 to 2002 [37]. Other works show how the number of patients with different combinations of chronic conditions is expected to increase in the future [38]. Moreover, the increasing prevalence of chronic disease among the near-elderly forecasts a gradual increase in morbidity among the elderly [22].

Individual chronic conditions and polypathology have been included in the model. However, the main limitation of the study is to build a morbidity projection from a fixed transition pattern corresponding to the period 2004–2007. More research on transition distributions and essentially large series of data are needed in order to break through the stationary assumption.

### Costs limitations and future research

Our model includes mean full costs from SSIBE corresponding to the year 2007. Such mean costs were aggregated by health status, age group and gender. Therefore, according to the CRG literature, the amount of variance explained by the model in respect to the potential realisations of costs was around 20% [19,33].

Since our article is devoted to introducing chronic conditions into lifetime healthcare costs estimates, other fundamental assumptions were deliberately assumed in their simplest form. However, there is a clear evidence of the importance of economic-related assumptions on forecasting exercises: the proportion of gross domestic product (GDP) spent on healthcare has a significant influence in forecasts [39]. At the same time, different studies reported the huge impact of innovation in future spending [22,40]. Moreover, the impact of changes in practices in future spending is estimated to be 3.8 times larger than the impact of demographic changes (e.g., ageing of the population) [41]. Consequently, more research is required in the area of costs as well. For example, the methodology of the Age Working Group considered aggregate scenarios on the future evolution of health expenditures [42]. Such scenarios were built from projections, assuming that unit costs evolve at the same rate as GDP per capita or per worker.

Due to the high concentration of costs in the last period of life, some authors considered health care expenditures to be a function of remaining life [43] or proximity to death instead of calendar age: the so-called red herring hypothesis [24]. However such studies are focused on an ageing population. Since the scope of our work is the whole life cycle, and chronic conditions and multimorbidity increase from early ages [44], calendar age was used instead of remaining life. Moreover, establishing current health conditional on the future (remaining life) would collide with the Markov property: health in the future (next year) is based on current health.

This study is limited in other ways as well. A larger population would allow us to describe individual morbidity and transitions under a more detailed scale: following the CRG hierarchy, ACRG3 (46 groups), ACRG2 (176 groups), ACRG1 (441 groups) or CRG (1,099 groups). Nevertheless, we are convinced that the six core health status groups describe chronic conditions and multimorbidity patterns reasonably well.

## Conclusions

Future morbidity and whole population costs can be reasonably predicted, combining stochastic microsimulation with a morbidity classification system. Potential ways of efficiency arose by introducing a time perspective to chronic disease management. The target of such new measures are people with chronic conditions and multimorbidity. Nevertheless, healthy individuals require specific policies.

Our model is just a first step. Considering stationary transitions and costs is a serious limitation. New sources of evidence on health state transitions and more sophisticated assumptions about future costs are needed.

### Endnotes

^a^According to the original definitions from the CRG manual [28], a healthy status is defined as follows: “A healthy status is identified by the absence of any primary chronic disease or significant acute episode diagnosis categories or episode procedure category. These individuals may have minor acute diagnosis present (e.g., upper respiratory infection, minor fractures, hernia, etc.) but are otherwise healthy. The healthy status also includes individuals who had no medical care encounters. CRGs are assigned hierarchically starting with most serious status, catastrophic, and going to the least serious, healthy Status 1 in the most recent six-month period. It is possible that in any population this includes a subset of individuals with chronic diseases who did not access the medical care system during the time period used to assign the CRG”.

^b^In the year 2007 CRG categories *2070 Pregnancy without Childbirth with Other Significant Illness*, *2080 Pregnancy and Childbirth with Other Significant Illness* and *2090 Pregnancy with Major Complications and Other Significant Illness* represented a 20% of acute processes for women included in the age group 15–24. Percentage values increased to 41% and 21% for women included in the age groups 25–34 and 35–44 respectively.

## Abbreviations

SSIBE: Serveis de Salut Integrats Baix Empordà; CRG: Clinical risk groups; POHEM: Canadian population health model; POPMOD: Population model; ICD: International classification of disease; AWG: Age working group; GDP: Gross domestic product.

## Competing interests

The authors declare that they have no competing interest.

## Authors’ contributions

MC designed and coordinated the study, provided cost information and developed simulation algorithms. PI conceived and promoted the idea. JC contributed to the conceptual design and coordination. IS performed the statistical analysis of simulation outputs and provided the artwork. JMI built the demographic-clinical database and performed the morbidity analysis. All authors contributed to the draft of this manuscript and approved the final version.

## Pre-publication history

The pre-publication history for this paper can be accessed here:

http://www.biomedcentral.com/1472-6963/13/440/prepub
